# Divalent cations are dispensable for binding to DNA of a novel positively charged olivomycin A derivative

**DOI:** 10.1371/journal.pone.0191923

**Published:** 2018-02-08

**Authors:** Artemy D. Beniaminov, Lyubov G. Dezhenkova, Olga K. Mamaeva, Anna K. Shchyolkina, Anna N. Tevyashova, Dmitry N. Kaluzhny, Alexander A. Shtil

**Affiliations:** 1 Engelhardt Institute of Molecular Biology, Russian Academy of Sciences, Moscow, Russian Federation; 2 Gause Institute of New Antibiotics, Moscow, Russian Federation; 3 Mendeleev University of Chemical Technology, 9 Miusskaya Square, Moscow, Russian Federation; 4 N.N. Blokhin National Medical Research Center of Oncology, Moscow, Russian Federation; 5 ITMO University, Saint Petersburg, Russian Federation; Helsingin Yliopisto, FINLAND

## Abstract

The current model of binding of the antitumor antibiotic olivomycin A (**1**) to GC-rich DNA regions presumes that coordination of the magnesium divalent cation with drug dimers is necessary for binding of **1** into the minor groove of the DNA duplex. Previously we have synthesized the derivatives of **1** termed ‘short acid’ (**2**) and its *N*,*N*-dimethylaminoethylamide (**3**). The latter compound demonstrated an improved tolerance *in vivo* compared to **1** and good therapeutic potency in animal models. We herein report that compound **3** is able to form stable complexes with DNA in the absence of Mg^2+^, in striking contrast to **1** whose binding to the DNA absolutely requires Mg^2+^. The mode of binding of **3** to DNA is similar in the presence or absence of Mg^2+^ as determined by circular dichroism. The affinity to DNA of **3** in Mg^2+^-free solution was similar to that of **1** or **3** in the presence of Mg^2+^ at low ionic strength. Non-electrostatic contributions to total free energy of binding of **1** and **3** to DNA were comparable for Mg^2+^-free complexes. Our data strongly suggest that electrostatic interaction of the positively charged **3** can compensate for the absence of divalent ions in complexes with DNA. This new property of the olivomycin A derivative expands the mechanistic knowledge of the modes of interaction with DNA of small molecular weight drug candidates.

## Introduction

**Fig 1:**
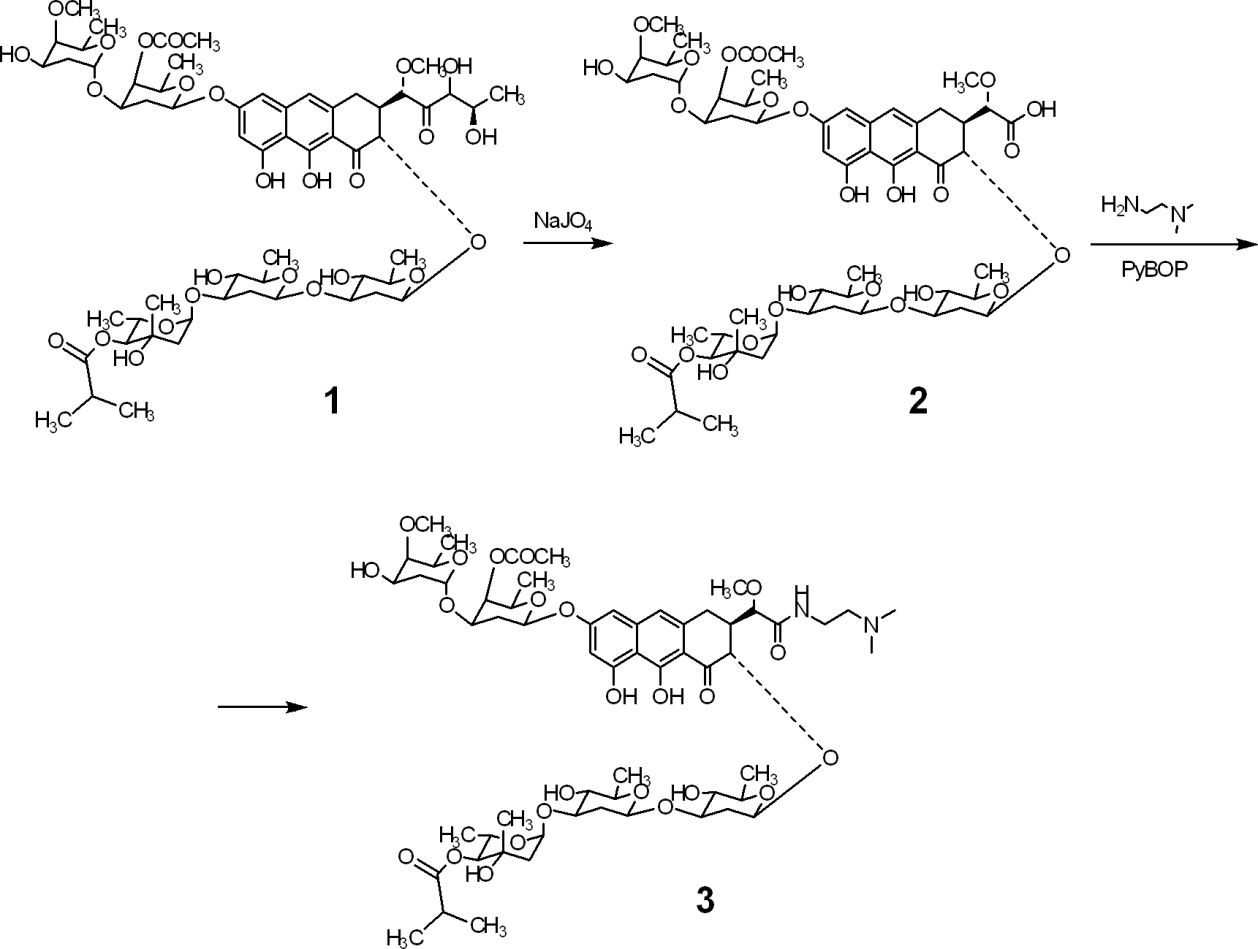
Chemical transformations of olivomycin A (1) into the ‘short acid’ (2) and *N*,*N*-dimethylaminoethylamide of the ‘short acid’ (3).

The aureolic acid family of antibiotics (chromomycin A3, mithramycin and olivomycin A) has long been considered a class of antitumor drug candidates. However, a strong cytotoxic potency of these agents is associated in general toxicity, thereby limiting their practical potential [1, 2]. Design of derivatives with retained antitumor potency and an improved tolerance *in vivo* is aimed at clinical applicability of this otherwise perspective chemotype. Various chemical transformations of olivomycin A (**1**; **Fig 1**) including the carbohydrate chains and the aglycon yielded the derivatives with different properties. In particular, among the congeners of **1** with a modified side chain of the aglycon a free acid (‘short acid’, compound **2**) was two orders of magnitude less cytotoxic than the series of amides of **2** [3]. Compound **3** (*L*-glutamate salt of *N*,*N*-dimethylaminoethylamide of **2**; **Fig 1**) carrying a positive charge at the tertiary amino group in the aglycon’s side chain demonstrated a high antitumor efficacy against the transplanted leukemia and melanoma at doses well tolerated by tumor bearing mice [3]. These results strongly suggested that compound **3** deserves further investigation as a drug candidate.

The DNA minor groove is a major intracellular target for aureolic acid derivatives. Thus far, target(s) other than DNA have not been identified for **1.** It is highly likely that binding of the drug to DNA is the major mechanism of cytoxicity. Indeed, the increased cytotoxicity of **3** was paralleled by a 6.4-fold bigger DNA binding constant compared to **2** [3]. Therefore, the drug:DNA complex formation can be a criterion for rational design of practically applicable derivatives of **1**.

Mechanisms of interaction of the aureolic acid derived antibiotics with DNA have been intensively studied by a plethora of methods including NMR and X-ray [4–9]. According to these studies, chromomycin A3 binds as a Mg^2+^-coordinated dimer to GC-rich sites in the DNA minor groove. The magnesium ion in the complex has an octahedral coordination where four oxygen atoms of chromomycin A3 are bound to Mg^2+^ and two water molecules comprise the fifth and the sixth ligand. Divalent cations are indispensable for the formation of stable drug-DNA complexes; Ni^2+^, Co^2+^ or Fe^2+^ demonstrated similar or even stronger effects than Mg^2+^ on complex formation [9]. We herein report that divalent cations are not required for the formation of stable complexes between the positively charged olivomycin A derivative **3** and DNA. Characteristics of the Mg^2+^-free complexes of **3** with DNA resemble those of the Mg^2+^-containing complexes. These data provide evidence for an alternative, i.e., cation independent, mode of interaction between the aureolic acid derivatives and DNA.

## Experimental

### General

All reagents were purchased from Sigma-Aldrich unless specified otherwise. Compound **3** was obtained according to **Fig 1** as described by us [3]. The synthetic route included oxidation of the aglycon’s side chain in **1** by sodium periodate. The resulting ‘short acid’ **2** was introduced into the amidation reaction with 2-aminoethyldimethylamine in the presence of benzotriazol-1-yl-oxytripyrrolidinophosphonium hexafluorophosphate. Compounds **1–3** were dissolved in dimethyl sulfoxide as 10 mM stock solutions and stored at 4°C. Aliquotes of **1–3** were added to aqueous reaction mixtures and immediately processed for the respective measurements (see below). The experiments were carried out in the binding buffer (BB: 13.7 mM NaCl, 0.27 mM KCl, 1 mM phosphate buffer pH 7.4) or BB-Mg (same solution supplemented with 5 mM MgCl_2_) at room temperature.

### Agarose gel electrophoresis

Compounds **1–3** were incubated with 0.2 μg of pUC19 plasmid (Thermo Fisher Scientific, USA) in BB or BB-Mg (total volume 15 μl, final DNA concentration 20 μM; bp) at 37°C for 30 min before loading the reaction mixture on a 1% agarose gel. The gels were run at 100 V for 1 h in 0.5x Tris base/boric acid/ethylenediaminetetraacetate (TBE) buffer (pH 8.3) and visualized after UV excitation on a ChemiDoc™ XRS+ System (BioRad). After the completion of electrophoresis the gels were stained with ethidium bromide (EtBr) for 15 min and visualized again. In some experiments EtBr was present in the gels and in the buffer during electrophoresis. Migration of free (unbound) compounds was tested in a 2.5% agarose gel in 0.2x TBE at 700 V for 3 min.

### Fluorescence titration

Fluorescence of **1–3** within 450–750 nm wavelength range was registered in a 140 μl quartz cell on a Cary Eclipse spectrofluorimeter (Varian) with excitation at 440 nm. The pUC19 plasmid (10–20 μM; bp) in BB or BB-Mg was treated with **1–3**. The time for drug-DNA complex formation was 20 min at each step. To determine the dependence of the binding constant on KCl concentration (10–100 mM) we used 5 μM of **1** and **3** in 20 mM Tris-HCl buffer pH 8.0 at 25°C. The concentration of the calf thymus DNA (ctDNA) varied within 2–50 μM (bp).

Values of binding constants were determined in Scatchard coordinates with fitting to McGhee-von Hippel equation *r*/*C*_*f*_ = *K*(1-*L·r*)^L^/(1-(*L*-1)*r*)^(*L*-1)^ where *C*_*f*_ is the concentration of free compound; *r* is average number of bound dimer molecules per one DNA base pair; *L* is the length of the compound on the DNA (considering 3 bp per dimer) [10, 11]. Gibbs free energy was determined with the equation Δ*G*° = *RT*ln*K*. Dependence of the binding constant on salt concentration was defined with the slope SK = ∂log*K*/∂log[KCl]. Using this slope the polyelectrolyte contribution to free energy of interaction can be calculated using the relation ΔG_PE_ = (SK)*RT*ln[KCl]. The non-electrostatic contribution into total energy Δ*G*_t_ was calculated as the difference Δ*G*_t_ = Δ*G*°- Δ*G*_PE_ [12]. Error values were estimated based on least-squares fits of data.

### Circular dichroism (CD) spectroscopy

CD measurements were performed on a Jasco-715 CD spectrometer using a quartz cell with a 10 mm optical path length. Spectra were obtained at a 1 nm bandwidth. Three scans were averaged. Compound **3** (20 μM in BB or BB-Mg) was titrated with ctDNA. CD spectra of DNA were not subtracted from the CD spectra of complexes due to their low amplitude.

## Results and discussion

### Role of Mg^2+^ in complex formation of compounds 1–3 with DNA

**Fig 2:**
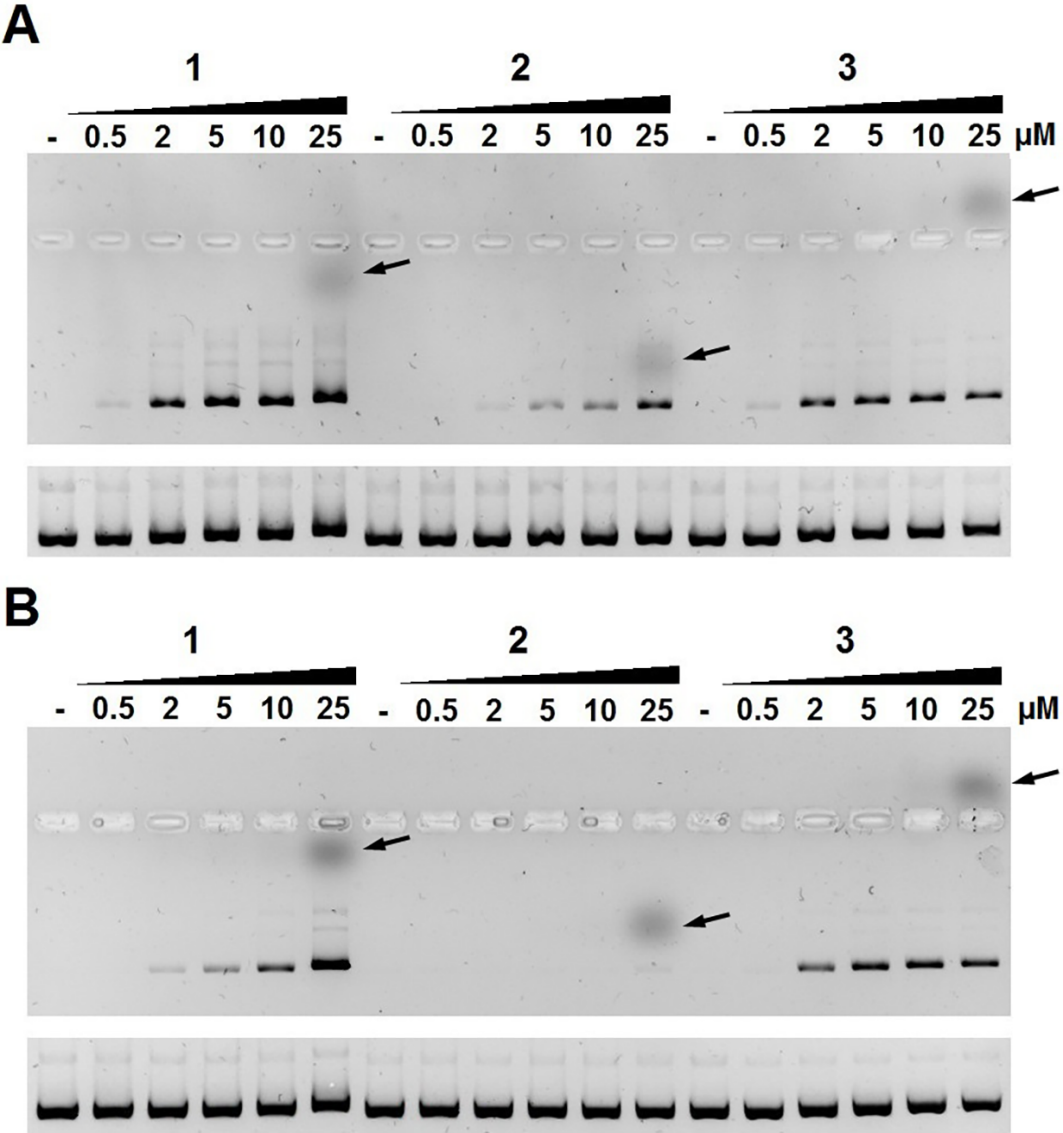
Binding of compounds 1–3 to the pUC19 plasmid DNA monitored by electrophoretic mobility in 1% agarose gel. The plasmid was incubated with the compounds at indicated concentrations (μM) in BB-Mg buffer containing 5 mM MgCl_2_ (A) or BB buffer (same buffer with no MgCl_2_) (B). Migration of the free compound is shown at the highest concentration (25 μM) for each drug (arrows). Bottom panels in A and B: electrophoresis with EtBr in the gel and in the running buffer.

We studied the binding of olivomycin A (compound **1**) and its structurally close derivatives **2** and **3** (**Fig 1**) to ctDNA by two independent methods: gel migration and fluorescence. The gel retardation assay can reflect the mass, the charge or the conformational changes of the plasmid DNA after the formation of stable complexes with the drug. Fluorescent properties of **1–3** allow for assessment of drug-DNA complexes by gel electrophoresis. **Fig 2A** (top panel) shows that the pUC19 plasmid incubated with **1–3** in the presence of Mg^2+^ retarded its migration in the agarose gel due to the formation of complexes with **1**, **2** or **3**. As expected, **1** and **3** demonstrated a higher affinity to DNA (manifested in a slower migration of the plasmid) than **2**. Staining of the same gel with EtBr and subsequent UV visualization (**Fig 2A**, bottom panel) also revealed a slightly retarded migration of DNA in complexes with **1** and **3** compared to free DNA, while no retardation was detectable after incubation of the plasmid with **2**. When the gels were run in the presence of a saturating amount of EtBr, the differential migration of the plasmid was more pronounced (**Figure 1 in S1 File**). This effect can be explained by replacement of the intercalator EtBr by the drug.

In striking contrast, when the drug-DNA binding experiments were performed in the buffer without Mg^2+^, the DNA binding affinities of **1** and **2** changed. The binding of **1** was somewhat weaker whereas **2** lost its ability to bind the plasmid. Importantly, the affinity of **3** to DNA remained unaffected (**Fig 2B**). These data indicated that retardation of the plasmid in complexes with **1** or **3** was observable even in the absence of Mg^2+^ ions. Of note, the titration of the plasmid reached saturation with or without Mg^2+^, since migration of unbound compounds was discernible at the highest drug concentration (spots at 25 μM). To confirm the observed migration of free **1–3**, these compounds were run separately (**Figure 2 in S1 File**). Differential migration of compounds largely reflects their electric charge in the electrophoretic buffer. Compound **1** demonstrated a low migration rate towards the positively charged electrode; this fact can be explained by a weak negative charge due to partial deprotonation of C9 phenolic hydroxyl group. Compounds **2** and **3** migrated in opposite directions indicating that at pH~8.3 **2** is negatively charged while **3** is positively charged. A negative charge of **2** is attributable to deprotonation of the carboxyl moiety whereas a positive charge of **3** is likely to be a result of protonation of the dimethylamino group.

**Fig 3:**
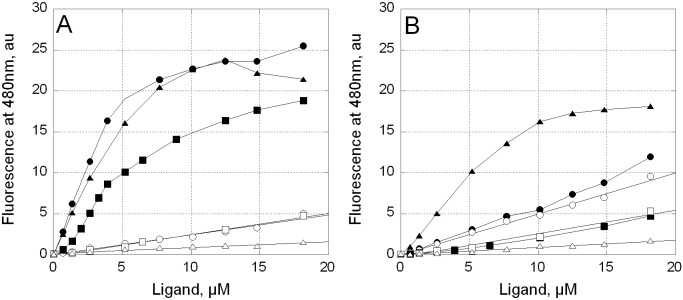
Fluorescence of free and bound to DNA of compounds 1–3. Comparison of fluorescence intensity of compounds **1** (circles)**, 2** (squares) and **3 (**triangles) in the presence (A) or absence (B) of magnesium cations. Open symbols, fluorescence of free (unbound) **1–**3; filled symbols, fluorescence of **1–3** in complexes with pUC19 DNA (10 μM; bp). Excitation: 440 nm, fluorescence detection: 480 nm.

**Fig 4:**
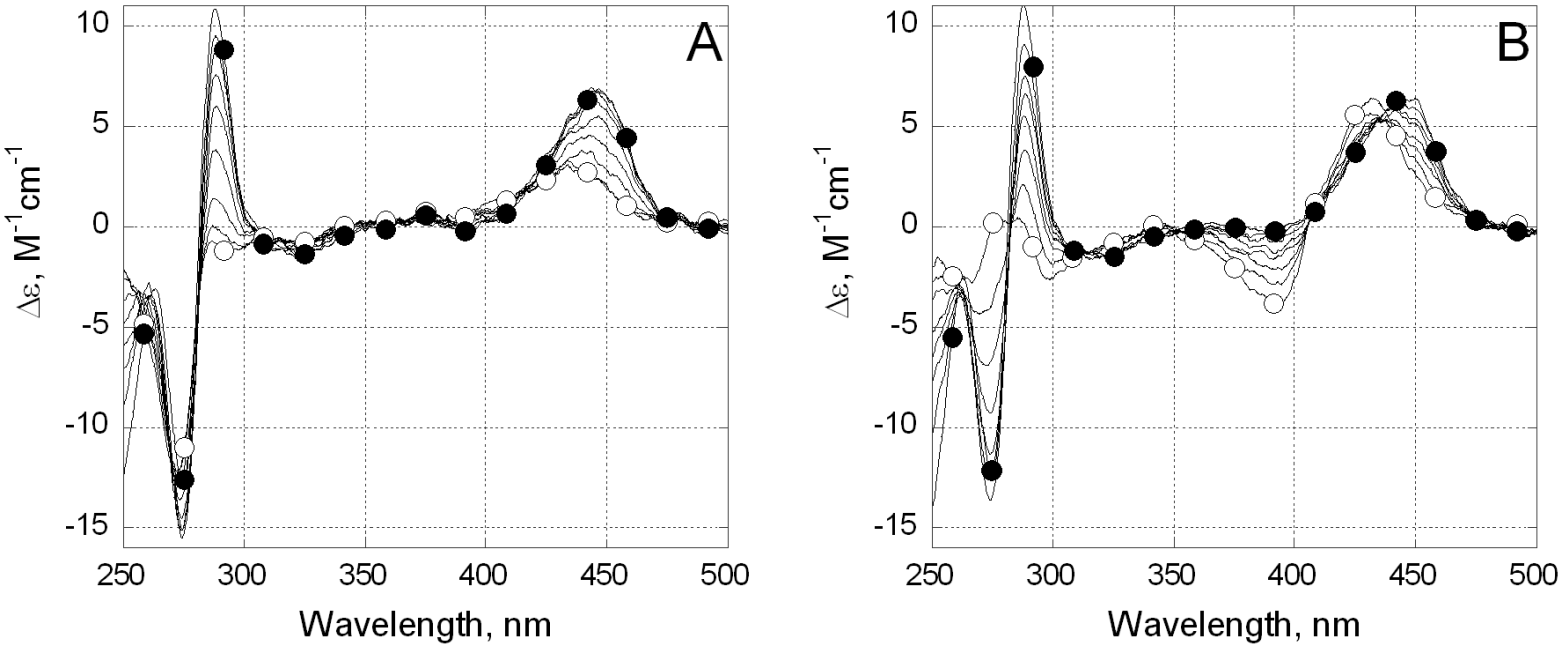
Changes of CD spectra of compound 3 upon binding to DNA. CD spectra of unbound compound **3** in the absence of DNA (open circles) and in complexes with DNA (filled circles) in BB-Mg (A) or BB (B). DNA concentration increased in the range 5–50 μM (bp), the concentration of **3** was 20 μM.

One may expect that differential binding of **1–3** to DNA in Mg^2+^ *vs* Mg^2+^-free conditions detected by in-gel migration should be independently proved in solution. We therefore studied the interaction of **1–3** with the plasmid DNA using optical methods. The binding of **1**–**3** to DNA can be conveniently registered in solution since fluorescence of unbound (free) and bound compounds differ significantly. We recorded the emission spectra at excitation wavelength of 440 nm for the increasing amounts of **1**–**3** added to the pUC19 plasmid in BB-Mg or BB (**Figures** 3 and **4 in S1 File**, respectively). Spectra of compounds in complexes with DNA were characterized by an increased fluorescence intensity with maximum at 540 nm and a ‘shoulder’ at 480 nm [13]. Fluorescence intensity at 480 nm, a signature of the drug-DNA complex, was used for plotting the binding curves in order to diminish the contribution of the unbound compound into fluorescence. The drug-DNA complex formation was characterized by the difference between the fluorescence intensity of unbound compounds and the fluorescence intensity of compounds in complexes with DNA (**Fig 3**, open and filled symbols, respectively). In the presence of Mg^2+^ the intensity of fluorescence of **1–3** bound to DNA increased significantly (**Fig 3A**). In BB (no Mg^2+^) fluorescence of free **1**–**3** increased linearly with a slight slope (**Fig 3B**). In Mg^2+^-free buffer the difference between the fluorescence of unbound *vs* DNA-bound **1** and **2** was very small (**Fig 3B**) indicating that **1** and **2** required Mg^2+^ for interaction with DNA. In contrast, fluorescence of **3-**DNA complex in BB elevated almost as sharply as in BB-Mg buffer (**Fig 3A and 3B**, filled triangles). Thus, fluorescence titration experiments revealed that **1**–**3** interacted with DNA in the presence of Mg^2+^ but only **3** formed stable complexes with DNA in the absence of Mg^2+^.

### Mg^2+^-free complexes of 3 with DNA

CD spectroscopy was used to characterize the Mg^2+^-free complexes of **3** with the duplex DNA. CD spectra of unbound **3** in BB and BB-Mg differed (**Fig 3A and 3B,** open circles). In BB, compound **3** showed a positive band near 440 nm, a negative one near 390 nm and weak bands in the UV region. The addition of Mg^2+^ ions significantly changed the CD spectra of **3**. The positive band at 440 nm reduced its amplitude, the negative band at 390 nm disappeared whereas a strong negative band at 270 nm emerged. These changes were associated with Mg^2+^ binding by free compound **3** (**Fig 4**, open circles). The addition of DNA to the solution of **3** changed CD spectra both in BB-Mg and in BB. In complexes with DNA, the CD spectra of **3** revealed a red shift to 450 nm of the long wavelength positive band, no negative long wavelength band around 390 nm, a strong positive UV band near 290 nm and a strong negative one at 270 nm (**Fig 4**, filled circles). Most importantly, CD spectra of **3** bound to DNA were strikingly close in the presence or absence of Mg^2+^. These results demonstrated a similar mode of complex formation between **3** and DNA regardless of Mg^2+^.

### Crucial role of electrostatic interaction in binding of 3 to DNA

**Fig 5:**
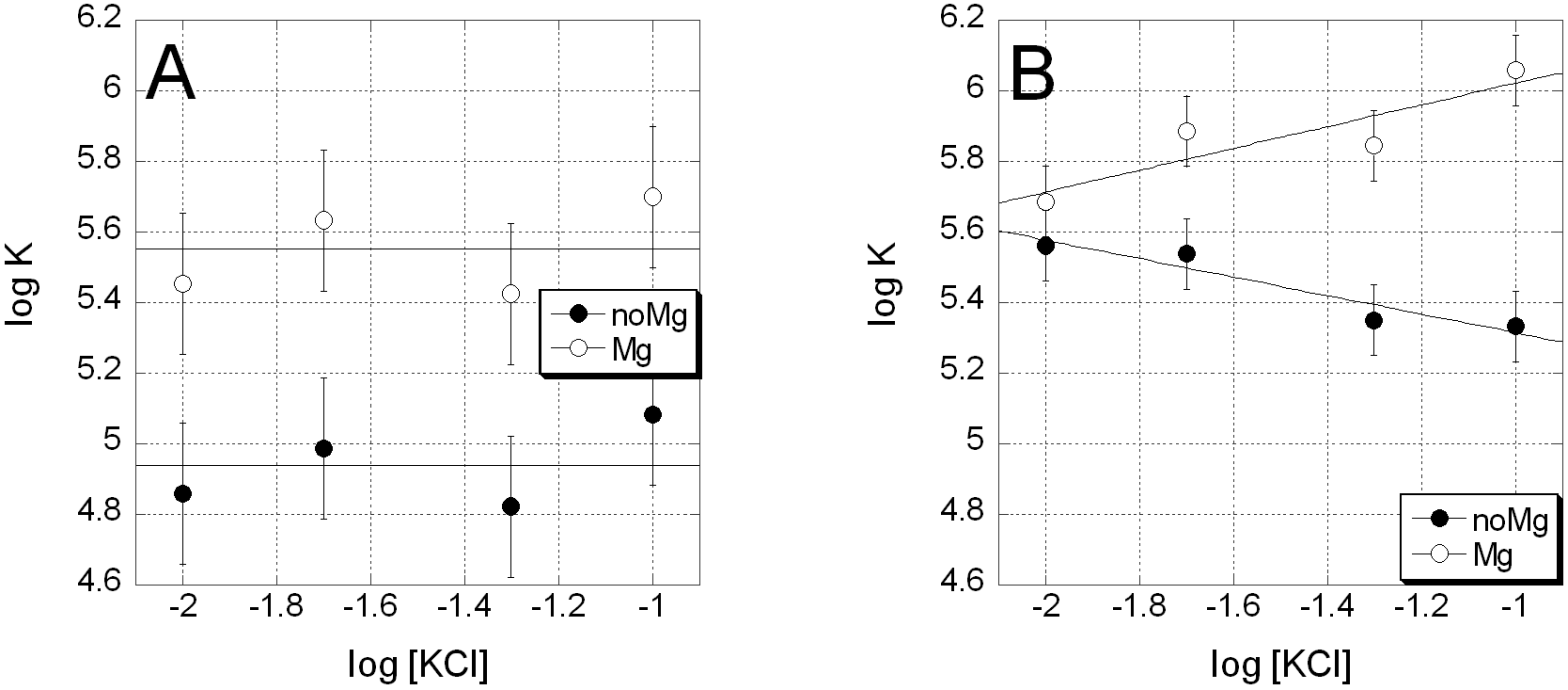
**Dependence of DNA binding affinity of compounds 1 (A) and 3 (B) on the ionic strength of solution.** DNA binding constants of **1** and **3** obtained in solutions that contained 5mM MgCl_2_ (filled circles) and no MgCl_2_ (open circles). Linear approximations shown by solid lines with the slope SK = ∂logK/∂log[KCl].

One may assume that the affinity of **3** to DNA depends on the ionic strength of the solution since the compound carries a positive charge. To address this question, we tested the influence of KCl (10–100 mM) on binding of **1** or **3** to the duplex DNA. The binding affinities were determined by titrating each compound with ctDNA and monitoring fluorescence. Typical fluorescence spectra and an isotherm are shown in **Figure 5 in S1 File**. The association constants (**Table 1 in S1 File**) were determined by fitting the data to McGhee-von Hippel equation (*L* = 3 bp per compound dimer). Values of the binding energy (**Table 1**) indicated a strong binding of **1** and **3** in the presence of Mg^2+^; however, only compound **3** was able to bind to DNA in the absence of magnesium cations in the solution. As expected, a negligibly low dependence of binding affinity on the solution’s ionic strength for the uncharged **1** was observed (**Fig 5A**). In contrast, the binding affinity of **3** highly depended on the ionic strength (**Fig 5B**). Normally, if electrostatic forces are predominant for the interacting molecules, an increased concentration of counterions reduces the electrostatic interaction, thereby weakening the binding. This was the case for interaction of **3** with DNA in the Mg^2+^-free buffer. In contrast, the increasing ionic strength stabilized the complex **3-**DNA in the presence of Mg^2+^. This phenomenon can be explained by the formation of dimers with an unfavorable geometry for DNA binding due to electrostatic repulsion of positively charged drug monomers. A fraction of the unfavorable dimers present in BB-Mg solutions may decrease upon increasing the ionic strength, thereby facilitating binding of 3 to DNA (Fig 4B, filled circles).

**Table 1:** Salt dependent thermodynamic parameters of binding of 1 and 3 to DNA calculated on the basis of fluorescence measurements.

Parameter	Compound 1		Compound 3	
MgCl_2_, mM	0	5	0	5
ΔG° _10mM KCl_, kcal/mol	-6.6±0.2	-7.5±0.3	-7.6±0.2	-7.8±0.2
SK = ∂logK/∂log[KCl]	0±0.15	0±0.15	-0.26±0.06	0.3±0.1
ΔG_t_ = ΔG-ΔG_PE_, kcal/mol	-6.6±0.2	-7.5±0.3	-6.9±0.2	-8.7±0.2

See ‘Experimental’ for details.

The electrostatic contribution to total free energy of interaction for **1** and **3** can be calculated from the salt dependence of the binding constant for these compounds [12]. The non- electrostatic contribution to total energy ΔG_t_ was determined as a salt independent component. Interestingly, ΔG_t_ in Mg^2+^-free solution for **1** and **3** were approximately the same (~ -7 kcal/mol). This result indicates that electrostatics is a major factor of differential interaction of **1** and **3** with DNA in the absence of Mg^2+^; in other words, partial positive charge of **3** compensates for the absence of divalent cations in complexes with DNA.

Thus, chemical modifications of olivomycin A that initially intended to decrease its toxicity and improve therapeutic efficacy yielded the derivative **3** with an alternative mode of interaction between the drug and the DNA. Our findings demonstrate that, while the affinity of the derivative to DNA is preserved, divalent metals are dispensable for drug-DNA complex formation at relatively low ionic strength of solution. In this scenario a coordination of drug dimers by divalent cations is not necessary: the non-coordinated drug can also form stable complexes with DNA.

## Conclusion

In this study we analyzed the mode of interaction with the duplex DNA of the antibiotic olivomycin A (**1**) and its two derivatives: a ‘short acid’ (**2**) and its *N*,*N*-dimethylaminoethylamide amide (**3**). We demonstrated a striking capability of compound **3** to form stable complexes with DNA in the absence of any divalent metal ions. The affinities of **3** to DNA in complexes with or without Mg^2+^ were similar. Modes of interaction of **3** with DNA in the presence or absence of Mg^2+^ were also similar as determined by CD. We suggest that this uncommon property of the aureolic acid congener results from partial positive charge bearing by the molecule.

## Supporting information

S1 File Supplementary data. (PDF)
