# Correction: Divalent cations are dispensable for binding to DNA of a novel positively charged olivomycin A derivative

**DOI:** 10.1371/journal.pone.0193630

**Published:** 2018-02-23

**Authors:** The PLOS One Staff

There are errors in the Funding section. The publisher apologizes for the error. The correct funding information is as follows: This study was funded by the Russian Science Foundation (project 16-14-10396) to DK. The funders had no role in study design, data collection and analysis, decision to publish, or preparation of the manuscript.
