# Supplementary material for: Divalent cations are dispensable for binding to DNA of a novel positively charged olivomycin A derivative
Source: PLoS One. 2018 Feb 8;13(2):e0191923. doi: 10.1371/journal.pone.0191923 (PMC5805246; doi:10.1371/journal.pone.0191923)
Supplement: S1 File — (PDF) [file pone.0191923.s001.pdf]

Supplementary data for

Divalent Cations Are Dispensable for Binding to DNA of A Novel Positively Charged Olivomycin A Derivative

Artemy D. Beniaminov[1], Lyubov G. Dezhenkova[2], Olga K. Mamaeva[1], Anna K. Shchyolkina[1], Anna N. Tevyashova[2,3], Dmitry N. Kaluzhny[1], Alexander A. Shtil[4,5]

[1]Engelhardt Institute of Molecular Biology, Russian Academy of Sciences, 32 Vavilov Street, 119991 Moscow, Russian Federation

[2]Gause Institute of New Antibiotics, 11 B. Pirogovskaya Street, 119021 Moscow, Russian Federation

[3]Mendeleev University of Chemical Technology, 9 Miusskaya Square, 125190 Moscow, Russian Federation

[4] N.N.Blokhin National Medical Research Center of Oncology, 24 Kashirskoye Shosse, 115478 Moscow, Russian Federation

[5]ITMO University, 49 Kronverksky Avenue, 197101 Saint Petersburg, Russian Federation

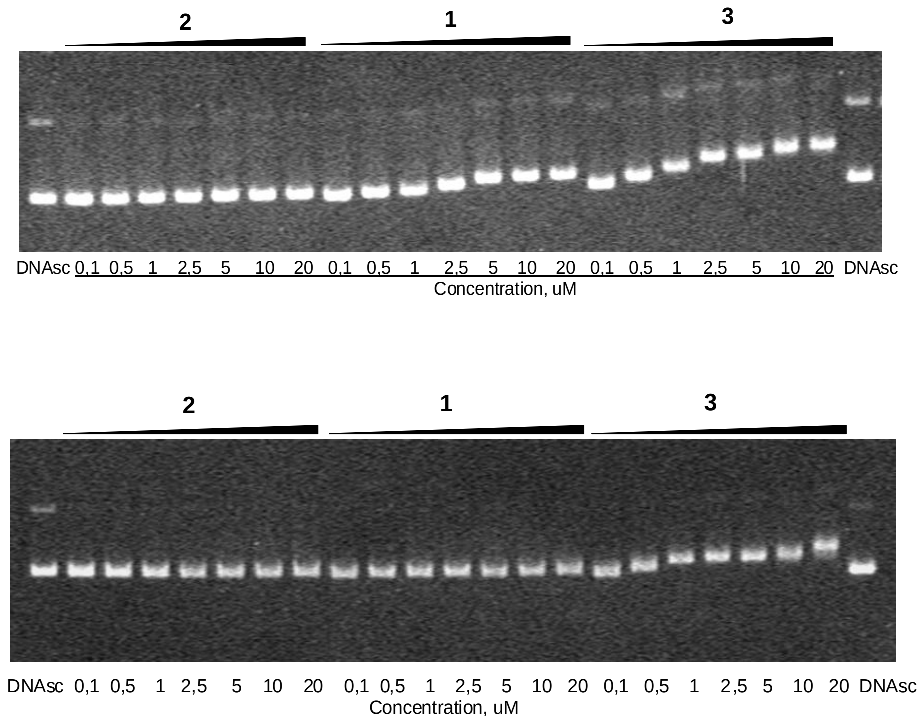

Figure 1. Effects of compounds 1-3 on electrophoretic migration of the pUC19 plasmid in EtBr containing gel. Compounds were incubated with the plasmid in BB-Mg (*upper panel*) or BB (*lower panel*) followed by electrophoresis in 1% agarose gel. EtBr was present in the gel and in the running buffer.

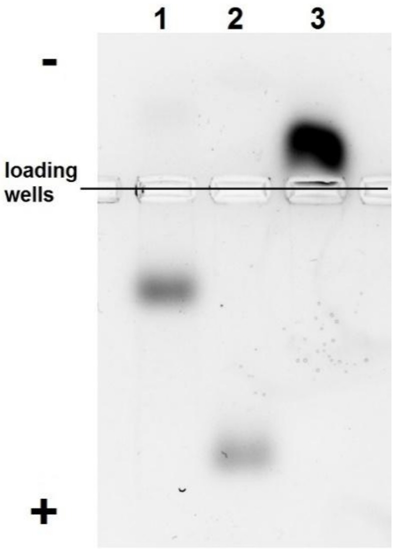

Figure 2. Electrophoretic migration of free (unbound) compounds 1-3 in 2.5% agarose gel (0.2x TBE, 700 V, 3 min).

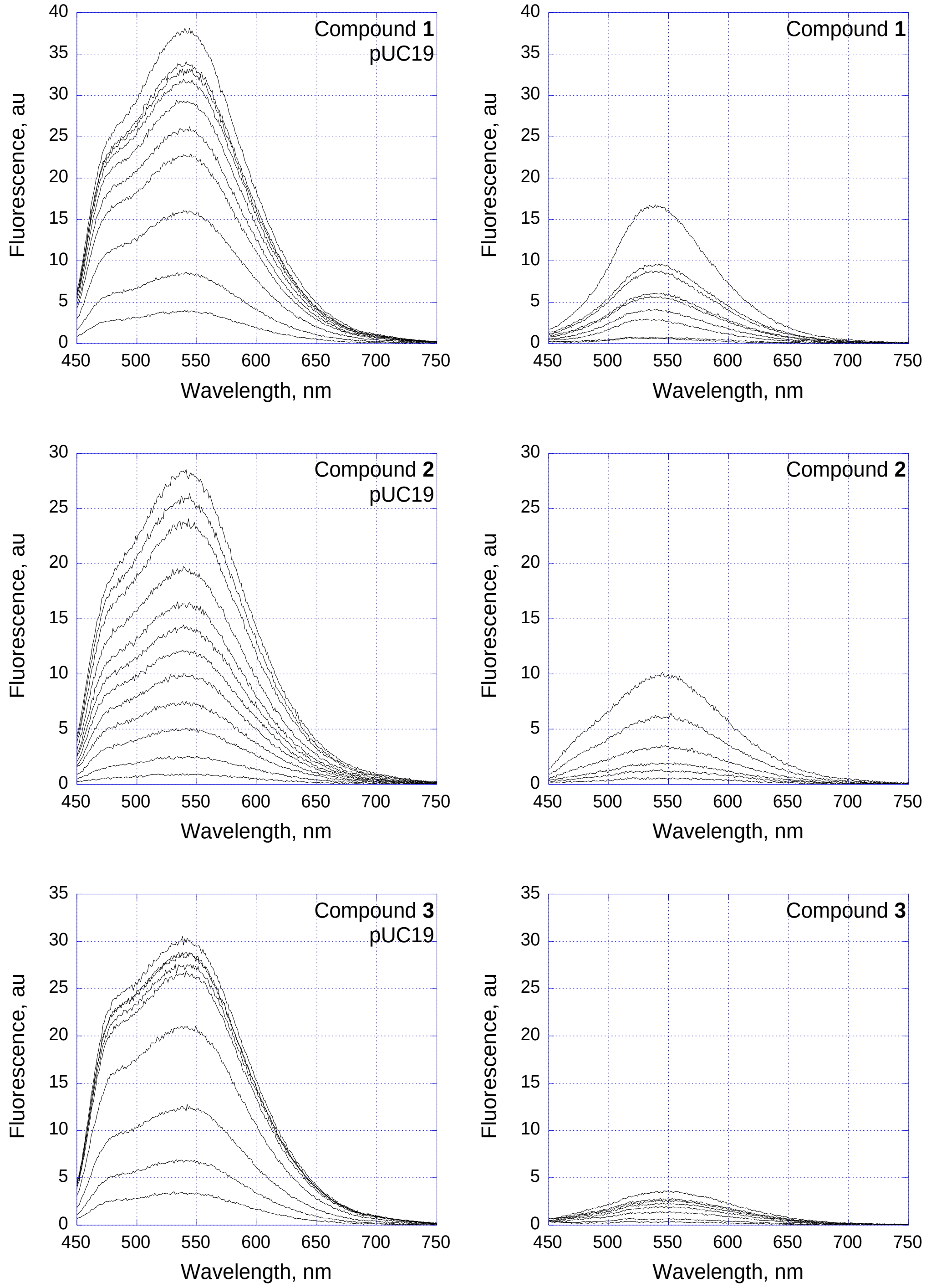

Figure 3. Fluorescence spectra of compounds 1-3 in BB-Mg with (*left panels*) and without (*right panels*) pUC19 DNA. DNA concentration was 10 μM(bp), compounds concentration increased from 0.5 to 18μM.

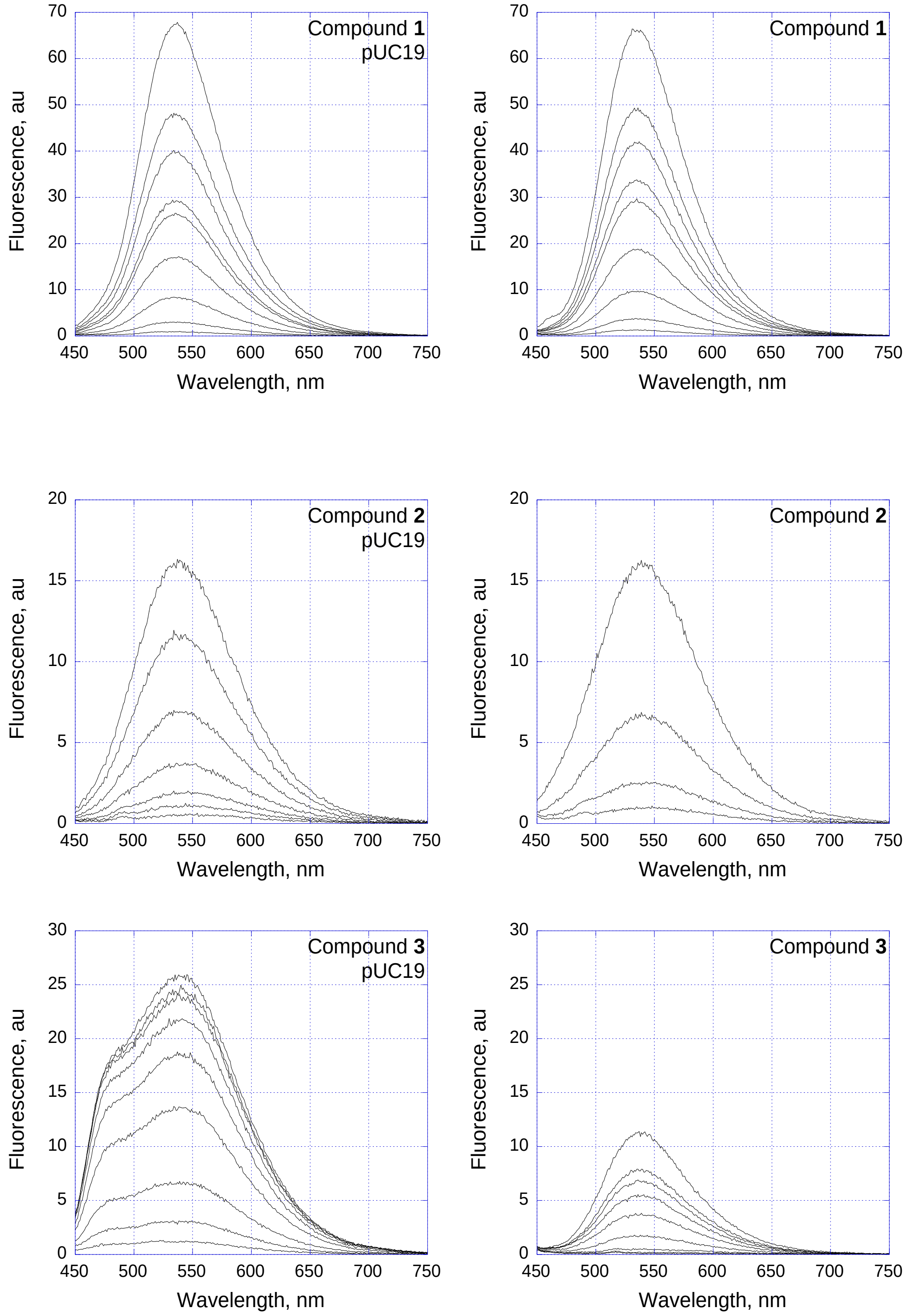

Figure 4. Fluorescence spectra of compounds 1-3 in BB with (*left panels*) and without (*right panels*) pUC19 DNA. DNA concentration was 10 μM(bp), compounds concentration increased from 0.5 to 18μM.

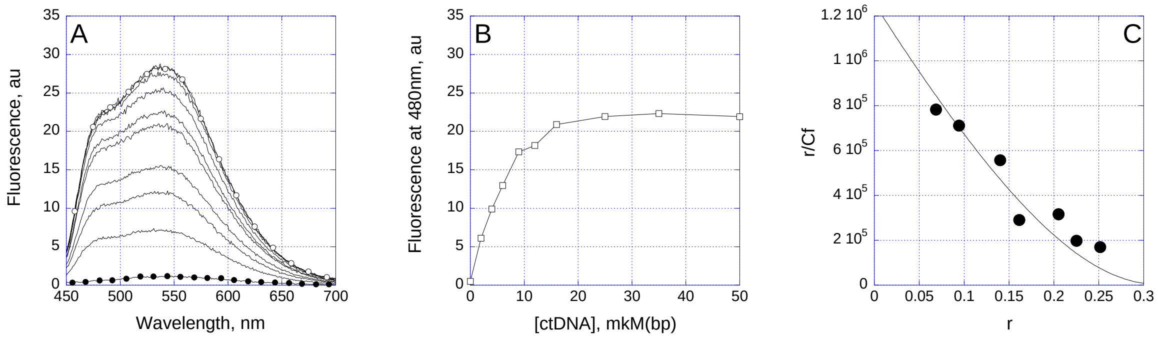

Figure 5. Fluorescence spectra (A), fluorescence intensity at 480 nm (closed circles, no DNA, open circles highest DNA concentration 50µM(bp)) (B) and binding isotherm (C) for compound 3 in 5 mM $MgCl_2$, 100 mM KCl solution.

Table 1. Salt dependence of DNA binding constants for compounds 1 and 3.

**[KCl], mM**	Compound **1**		Compound **3**	
	**$MgCl_2$, mM**			
	0	5	0	5
10	0.7±0.4*	2.8±0.2	3.6±0.4	4.8±0.3
20	1.0±0.5	4.3±0.2	3.5±0.4	5.8±0.2
50	0.7±0.4	2.7±0.1	2.2±0.4	7.0±0.3
100	1.2±0.6	5.0±0.3	2.2±0.3	11.0±0.4

*K, 10^5 M^{-1} determined from fluorescence data.
